# The Biopontis Alliance Rare Disease Foundation (BARDF) – an innovative model for early stage rare disease therapy financing and development

**DOI:** 10.1186/1750-1172-9-S1-O18

**Published:** 2014-11-11

**Authors:** Erik T Tambuyzer, Barbara L Handelin, Richard A Basile, Marlene E Haffner

**Affiliations:** 1 BARDF Board of Directors, Belgium; 2 BARDF, Philadelphia, PA, USA; 3 BARDF, Raleigh, NC, USA; 4 BARDF Board of Directors, USA

## 

The right therapy for the right patient at the right time is the most important approach for the provision of proper health care. The field of rare diseases is an excellent example, with patients taking a more central role in the discovery and development of treatments for their disease. Limited budgets coupled with high failure rates of medicine development lead us to public-private partnerships as a model to move forward and reduce cost; no stakeholder alone has all knowledge and expertise.

The newly founded BioPontis Alliance Rare Disease Foundation presents a scientific and economic partnership model designed to unleash the potential to find cures for rare (pediatric) diseases. The non-profit Foundation has developed an innovation joint venture business and scientific development structure to fill the gap between discovery science and the biomedical industry. The Foundation will establish scientific bridges across the discovery/preclinical development gap as a partner to independent academic and/or patient organizations to develop medicine candidates. The BioPontis Alliance Rare Disease Foundation intends to also establish a financial bridge between the cures ‘push’ of philanthropic resources and the commercial ‘pull’ of private sector.

The Foundation will provide a professional drug discovery operation in which the necessary scientific expertise for each product/discovery initiative can be selected and applied to each program. It has a virtual network of contract research companies and academic institutes where development work can be conducted as well as bringing central project management and industry experienced managers to each initiative. With novel licensing practices, the Foundation financially honors the seeding contributions of originating scientists, their host institutions and/or the Patient organization partner. Licensing agreements have already been signed with over 12 leading US academic institutions/hospitals, offering the originators a pro rata share of revenues that are generated from the full intellectual property estate, thus removing many of the misalignments of interests in traditional licensing and sponsored research agreements. Focus on a single goal of creating potential treatments for patients is therefore assured across the BARD Foundation’s model.

The BARD Foundation is currently setting up financing and is seeking donations for its operations. While operating for a global patient community and market, we seek contacts with potential financial, scientific and patient organization partners in Europe and other parts of the world.

